# The Presence of Molds and Their Secondary Metabolites in Purple Coneflower-Based Dietary Supplements (*Echinacea purpurea* (L.) Moench)

**DOI:** 10.3390/toxins14090607

**Published:** 2022-09-01

**Authors:** Gabriela Pilarska, Magdalena Twarużek, Iwona Ałtyn

**Affiliations:** Department of Physiology and Toxicology, Faculty of Biological Sciences, Kazimierz Wielki University, 85-064 Bydgoszcz, Poland

**Keywords:** dietary supplements, mycotoxins, mold contamination, *Echinacea purpurea*

## Abstract

Purple coneflower (*Echinacea purpurea* (L.) Moench) is a plant in the family *Asteraceae*, mainly grown in North America. *Echinacea purpurea* has been used in conventional medicine. The plant has immuno-stimulating and antibacterial properties, but neither mold contamination nor a mycotoxin presence have been evaluated. Our goal is to determine the degree to which molds and mycotoxins contaminate dietary supplements based on purple coneflower distributed on the Polish market. We analyzed 21 samples divided into four groups: sachets (*n* = 5), dry raw material (*n* = 3), capsules (*n* = 9), and tablets (*n* = 4). The mycological analysis of dietary supplements shows that the average number of molds is 1012 cfu/g, and the most common molds are *Aspergillus* spp., *Phoma* spp. and *Eurotium* spp. The mycotoxins most common in the samples are ZEN (18/21), DON (5/21) and T-2 toxin (3/21).

## 1. Introduction

*Echinacea purpurea* (L.) Moench is a member of the family *Asteraceae* and originates from North America, where it was used by Native Americans as a medicinal plant. The Comanche tribe used coneflower for toothaches and sore throats, while the Sioux tribes used it for rabies and inflammation [1,2]. European settlers brought the plant to Europe and learned to grow and use it. In Germany, England and France, coneflower did not gain popularity until the 1920s and 1930s, and in Poland not until the early 1990s [1]. Currently, preparations containing purple coneflower are used as herbal medicines and dietary supplements around the world [3]. *Echinacea purpurea* contains glycosides, flavonoids (free and glycoside-bound), ethereal oil, polysaccharides, active polyacetylenes, pyrrolizidine alkaloids, alkylamines and alkylamides [4]. The root of *Echinacea* contains chlorogenic acid, inulin, glycoproteins, cynarins and amine compounds, among others. These compounds stimulate the secretion of gastric and intestinal juices, boost regenerative processes, and have an antipyretic effect. The active ingredients of coneflower also accelerate metabolism and have antifungal and diastolic functions [5]. Previously [6,7,8], purple coneflower extracts were shown to have anxiolytic, antidepressant and cytotoxic effects, and to have anti-viral effects against influenza and corona viruses.

Herbaceous plants such as purple coneflower are used as daily products or as raw materials in the pharmaceutical industry to produce dietary supplements. These products are classified as foodstuffs [8,9]. Therefore, they are not subject to registration or to clinical trials, as is the case with medicines or pharmaceutical products [10]. Dietary supplements marked in the Republic of Poland must comply with the Food and Nutrition Safety Act of 25 August 2006 (Journal of Laws of 2010 No. 136, item 914 as amended) [11] and the Regulation of the Minister of Health concerning the Composition and Labelling of dietary supplements of 9 October 2007 (Journal of Laws of 2007 No. 196, item 1425 as amended) [11], as well as the Regulation of the Minister of Agriculture and Rural Development of 10 July 2007 concerning the labelling of foodstuffs (Journal of Laws of 2007 No. 137, item 966, as amended) [11]. The purple coneflower extract used to manufacture dietary supplements has a high risk of environmental contamination. The risk depends on the place of cultivation, weather conditions and the harvest time. Failure to comply with good manufacturing practices (GMP) and good hygienic practices (GHP) at various stages of production, i.e., storage, processing and the final product distribution, may also increase the risk of environmental contamination [11]. Thus, both desirable and undesirable substances may be present at varying levels in different parts of the plant, especially in extracts. Herbs, medicinal plants and dietary supplements may contain dangerously high levels of mycotoxins, such as aflatoxin (AF), ochratoxin A (OTA), fumonisins (FB), zearalenone (ZEN) and A- and B-trichothecenes, which may pose a threat to human health [12,13,14]. 

The United States Food and Drug Administration (FDA) classifies botanical products as drugs or dietary supplements on the basis of their end use. According to the FDA, in order to be marketed a drug must have an approved marketing application. Dietary supplements, on the other hand, are regulated by the Dietary Supplement Health and Education Act of 1994 [15]. To ensure the effective protection of public health, products that contain contaminants exceeding allowable levels should not be allowed on the market. In particular, a number of dietary supplements should be more accurately labeled for mycotoxicological and microbiological contamination. To ensure uniform implementation of the provisions on maximum levels of contaminants, the competent authorities must apply the same criteria for sampling and for analytical testing throughout the European Community (Commission Regulation (EC) No. 1881/2006 of 19 December 2006)). 

The objective of this study is to assess purple coneflower dietary supplements available on the Polish market for contamination with common mycotoxins. Our working hypothesis is that these supplements would be contaminated, but the level of contamination is unknown. This work advances the field by determining whether mycotoxin contamination of purple coneflower dietary supplements is high enough to pose a threat to human health.

## 2. Results 

### 2.1. Mycological Analysis of Dietary Supplements Based on Echinacea purpurea

The results show that the mean was 1108 cfu/g in the samples in which molds were accounted for 1012 cfu/g, which represent 16/21. The mycological analysis shows that 17/21 dietary supplements were contaminated by different species of molds. Yeast was accounted for in 7/21 of the samples, with a mean 96 cfu/g (Table 1).

**Figure 1:**
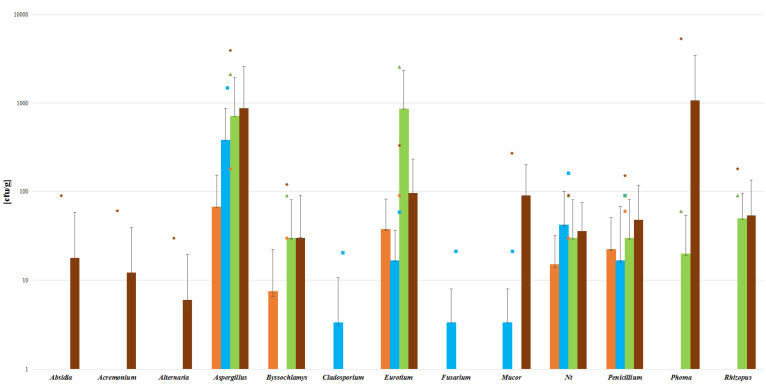
Parameters for mold contents in different forms of dietary supplement samples. ut-unidentified strains. (Bars indicate means, lines indicate SD and small symbols indicate the maximum level of mycotoxin. Colors indicate the source, with for orange is for tablets (*n* = 2/4), blue for capsules (*n* = 6/9), green for dry raw material (*n* = 3/3), and brown for sachets (*n* = 4/5).

An analysis of the contamination of the material tested for the presence of specific types of mold shows that of the 21 of tested samples, 12 were contaminated with *Aspergillus* spp. In 8 of the 21 samples, contamination with *Eurotium* spp. and *Penicillium* spp. was detected. The presence of this fungus is not a coincidence, since it is thermophilic and any treatment process may not have managed to remove it. *Penicillium* spp. may have appeared with the late harvest of the products, as they usually pore the highest amount of spores in spring or winter. The dietary supplements tested were available in four forms with various method of processing. These forms were tablets, capsules, sachets and dried raw material. Another part of the analysis was an assessment of the contamination with regard to the different forms of the dietary supplements. Raw materials were found to be the most contaminated form (3/3), followed by sachets (4/5), then capsules (6/9). Since these three forms are the least processed, the levels of mold contamination found in them may result from poor storage or incorrect handling. The least contaminated form were tablets (2/5) (Figure 1). 

### 2.2. Mycotoxicological Analysis of Dietary Supplements Based on Echinacea purpurea

**Figure 2:**
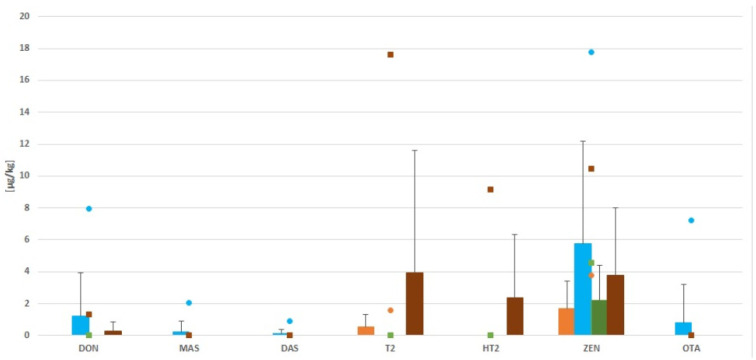
Levels of mycotoxin contamination in all samples. (Bars indicate means, lines indicate SD, and small symbols indicate the maximum level of mycotoxin. Colors indicate the source, with orange is for tablets (*n* = 2/4), blue for capsules (*n* = 6/9), green for dry raw material (*n* = 3/3), and brown for sachets (*n* = 4/5).

The mycotoxicological analysis of dietary supplements based on *Echinacea purpurea* showed that 18 of 21 samples were contaminated with mycotoxins. ZEN (18/21), DON (5/21) and T-2 (3/21) occurred in the highest frequency. The mean levels of these mycotoxins were 4 µg/kg, 1 µg/kg and 1 µg/kg, respectively. Ochratoxin A contaminated only one sample. Such levels of mycotoxins may result from early fungal contamination of plants growing in the field, and be further exacerbated by incorrect storage. Particularly dangerous conditions in this regard are high humidly and temperatures favorable for mold growth, which can produce mycotoxin biosynthesis. AF, NIV and 3ADON were not detected in the tested samples (Figure 2). 

The analysis conducted shows that the level of mycotoxin contamination of the various forms depends on the method of processing. All samples of dry raw material and sachets contained mycotoxins, while seven/nine samples of capsules and three/four tablets were contaminated by mycotoxin. As such, it can be concluded that the more the product is processed, the less contamination it contains; however, it is not completely free of them. Heat treatment carried out in conditions above 18% humidity and temperatures above 25 °C encourage fungal growth and mycotoxin production. Of all the mycotoxins that were analyzed, ZEN was found in all of the sachets and raw material samples. Capsules, on the other hand, had seven/nine samples contaminated, and the maximum concentration of ZEN was 17.8 µg/kg in this form. Ochratoxin A was detected in only one capsule, at the level of 7.21 µg/kg. (Figure 2).

The samples were also analyzed for mycotoxin co-occurrence. All four mycotoxins co-occurred in 11/21 samples, while three mycotoxins were found in 6/21 samples and two mycotoxins were found to co-occur 2/21 samples. Co-occurrence of mycotoxins in the same product increases the risk of toxin activity in supplement consumers.

## 3. Discussion

Raw materials used for the production of foodstuffs are contaminated with molds and their secondary metabolites, which poses a risk to the health of consumers. It is particularly dangerous when the product in question is a dietary supplement, which are commonly used in daily basic diets. Unfortunately, due to the lack of regulations of dietary supplements, most of these products are not tested, and their safety is poorly monitored. Dietary supplements contaminated at high levels with molds and their secondary metabolites (mycotoxins) which may pose a threat to human health [16,17,18,19,20]. 

Our analyses of *Echinacea purpurea* dietary supplements confirm that the available products are contaminated with molds and their secondary metabolites. However, it is not possible to compare our analyses with those of the herbal plant and extract itself. Thus, it is difficult to fully assess contamination, and one must instead assess dietary supplements based on plant extracts, herbs or spices. 

Previous analyses of dried medicines and medicinal plants showed contamination with molds such as *Aspergillus* spp., *Fusarium* spp., *Penicillium* spp., *Mucor* spp. and *Rhizopus* spp. [21,22]. Coffee beans were mainly contaminated with strains of *Aspergillus* spp. (63%) and *Penicillium* spp. (25%), while green coffee extracts were commonly contaminated by *Penicillium* spp. (72%) [23]. The highest OTA level was found in instant coffee (4.79 µg/kg) [24]. In sachets of herbal teas, strains of *Aspergillus* spp. (42%), *Penicillium* spp. (41%) and *Fusarium* spp. (26%) were the most common [25]. In analyses of green tea and royal jelly for pesticides and mycotoxins [26], only 1/10 green tea test samples was contamination with AFB_1_. 

The moldy product problem is not limited to medicinal plants and drinks, but also includes dried spices, with some surveys [27] finding filamentous fungal contamination in all tested products. The presence of mold in the dried product can result from harvesting raw material of poor quality which is already mold infested at the growth stage in the field, or from problems during storage. For example, in ginger [28], more AF and OTA were detected in the samples collected during in the rainy season (13 and 5.1 µg/kg) than in the dry season (1.18 and 2.76 µg/kg). AFB_1_ in 7/31 samples collected during the rainy season exceeded the European Union limit of 5 µg/kg. 

Herbs and herbal products are widely used in food and pharmaceutical industries and should be systematically controlled. Dietary supplements may be composed of multiple components, but if only one component is of poor quality, it still can reduce the quality of the final product [29,30,31,32,33]. Results can vary widely, and in some cases plant raw material contamination with aflatoxins never exceeded the LOD [34]. In milk thistle [35], the most common mycotoxins were HT-2 (92%), T2 (96%), DON (81%) and ZEN (89%). We found similar levels of ZEN contamination in the present study of purple coneflower extracts. Our samples were not contaminated by AF, and the amounts of OTA detected were not large. 

## 4. Conclusions

Herbal plants have been used for their preventive and medicinal properties since ancient times. However, plant raw material for the production of dietary supplements is susceptible to environmental contamination. In the analysis conducted the final product was studied, even though contamination can occur at any time from the growth of the plant in the field, through harvesting, and during processing, storage and transportation. Plants can be infected by more than one mold, and one species can produce more than one mycotoxin, so products may be contaminated with many different types of mycotoxins. This affects the material’s quality, safety and efficacy. Mycotoxins pose a risk to consumers’ health. Despite the presence of mycotoxins in the samples tested, the exposure of consumers’ due to ingestion of mycotoxins remains relatively low (less than 1%), and this does not pose a risk to the health of the consumers. As the consumption doses of dietary supplements are realistically low, the quantitative daily intake of mycotoxins will also be low. However, the increase in demand for these products requires that the product be free of mycotoxin levels. The lack of effective monitoring at each stage of production (harvesting, storing, processing and distribution) exposes consumers to contaminated products which can adversely affect their health. 

Dietary supplements should be a safe alternative to pharmaceutical products, and the risk of contamination should be non-existent. Although our findings are based on a small number of samples that were available on the Polish market, they show the presence of fungi and mycotoxins at a low level. However, their presence in herbal products should be used as an incentive to improve food quality and safety by implementing GACP and GMP at every stage of production.

## 5. Materials and Methods

### 5.1. Materials

The testing material consisted of 21 samples of dietary supplements based on purple coneflower (*Echinacea purpurea*) in four forms: sachets (*n* = 5), dry raw material (*n* = 3), capsules (*n* = 9) and tablets (*n* = 4). The formulations were purchased in pharmacies and herbal stores in Poland. The products came from different manufacturers.

### 5.2. Methods 

#### 5.2.1. Mycological Analysis

Material with a minimum weight of 5 g was finely ground and mixed. The prepared sample was placed in a sterile Stomacher-type homogenizer bag (BagMixer 400, Interscience, SAINT-NOM la Bretèche, France) weighing 5 ± 0.2 g. 

Samples were suspended in 45 ± 2% mL of sterile dilution fluid prepared according to PN EN ISO 6887-1, July 2000, consisting of: enzymatic casein hydrolysate—1 g, sodium chloride—8.5 g and distilled water—1000 mL, pH 7.0 ± 0.2, and homogenized for 90 s. The samples were resuspended in 45 ± 2% mL of sterile dilution fluid, prepared according to PN EN ISO 6887-1, July 2000, consisting of: enzymatic casein hydrolysate—1 g, sodium chloride—8.5 g and distilled water—1000 mL, pH 7.0 ± 0.2, and homogenized for 90 s. The total number of fungi was determined according to ISO 7954, September 1999, with modifications (1 mL surface culture, 0.1 mL in triplicate). The homogenized initial suspension (material a diluted 1:10) was diluted. The surface culture according Koch was performed on YGC (yeast extract, chloramphenicol, glucose) medium with the following composition: yeast extract—5 g, chloramphenicol—0.1 g, glucose—20 g, agar—15 g, distilled water—1000 mL, pH 6.6. The incubation was carried out for 5 to 7 days (10–14 in the case of filamentous fungi) at the specified temperature of 25 °C ± 1 °C. At the end of the incubation period, colonies were counted with a range not exceeding 10 to 100 colonies per gram. Separation into molds and yeasts was done on the basis of microscopic preparations. The results were expressed as the number of colony-forming units per gram of each sample [cfu/g] of fungi (filamentous fungi plus yeasts). Direct microscopic examination of colonies cultured from YGC agar was carried out on wet Amman’s lactophenol medium, according to the Polish Standard PN R-64791:1994 (1994) (Amman’s lactophenol: crystalline phenol—10 g, lactic acid—10 g, glycerol—20 g, distilled water—10 mL, methylene blue—0.5 g per 100 mL of lactophenol). Determination to genus was made using the Food and Indoor Fungi (Samson R.A.; Houbraken J.; Thrane U.; Frisvad J.C.; Andersen B) and Fungi and Food Spoilage (Pitt J.I.; Hocking A.D) keys.

#### 5.2.2. Mycotoxicological Analysis

##### Extraction of Aflatoxin 

First, 25 g of the sample was weighed into a 100 mL conical flask. Then, 2.5 g of NaCl was added. Next, 50 mL of MeOH:H_2_O mixture in a ratio of 80:20 was added. The whole mixture was homogenized for 1 min. The extract was filtered through a fluted filter (VICAM Fluted Filter Paper No. 31240, Milford, MA, USA) into a clean and sterile beaker. In another beaker, 40 mL of water was measured, then 10 mL of extract was added with thorough mixing. The diluted extract was filtered through a fine fiber filter (VICAM Microfibre Filters 1.5 µm No. 31955, Milford, MA, USA). The safety buffer was drained from the AflaTest^®^ column (Vicam, Watertown, MA, USA) without drying 10 mL of filtered, diluted extract was passed through the column. The column was then dried by passing air through it. The column was washed twice with 10 mL of H_2_O. Aflatoxins was eluted with 1 mL of MeOH solution by passing the solution very slowly, and in between the column was dried with air. The eluate was collected in a vial, and about 5 mL and 1 mL of H_2_O was added. The whole mixture was mixed using vortex (Heidolph Instruments GmbH & C, Schwabach, Germany).

##### Extraction of Ochratoxin A

A total of 25 g of the well-ground sample was weighed into a 100 mL conical flask. Then, 50 mL of can:H_2_O was added in a ratio of 60:40. The whole material was homogenized for 2 min. The extract was filtered through a fluted filter (VICAM Fluted Filter Paper No. 31240, Milford, MA, United States, Massachusetts) into a clean and sterile vessel. To another beaker, 55 mL of PBS (phosphate buffer, Merck KGaA, Darmstadt, Germany) was measured and 5 mL of supernatant was added. The diluted extract was filtered through a fine fiber filter (VICAM Microfibre Filters 1.5 µm No. 31955, Milford, MA, United States, Massachusetts) and 48 mL of the filtrate applied to an OCHRAPREP^®^ column (Rhone Diagnostic Technologies Ltd., Glasgow, UK). The extract was passed by gravity. After passing the extract, the column was dried with a stream of air. The column was washed with 20 mL of H_2_O, after which it was also was dried with air. Ochratoxin A was eluted with 1.5 mL of a solution of MeOH:CH_3_COOH in a ratio of 98:2. The solution was passed very slowly (1 drop/min) or by gravity. The column was then air dried and 1.5 mL of H_2_O was passed through. The eluate was collected in a vial of approximately 5 mL. The sample was mixed using vortex (Heidolph Instruments GmbH & C, Schwabach, Germany). 

##### Extraction of Trichothecenes and Zearalenone 

Extractions of trichothecenes and zeralaenone were performed on Bond Elut^®^ (Mycotoxin Agilent, Santa Clara, CA, USA). A total of 12.5 g of the well-ground sample was weighed into a 100 mL conical flask. Then, 50 mL of an ACN:H_2_O mixture 80:20 was added. Subsequently, the mixture was homogenized for 3 min. The extract was filtered through a fluted filter (VICAM Fluted Filter Paper No. 31240, Milford, MA, United States, Massachusetts) into a 100 mL beaker. A total of 40 µL of ZAN (^13^C-zearalenone solution (c = 1000 ng/mL)) standard solution and then 4 mL of filtered extract was added. The extract was filtered into a clean 5 mL vial. Internal standard solutions of 50 µL were added to the 5 mL vial (^13^C-DON (c = 2500 ng/mL), ^13^C-T2 (c = 250 ng/mL) and ^13^C-HT2 (c = 250 ng/mL)). The whole was evaporated to dryness using a nitrogen stream. To the dry residue was added 495 µL MeOH:H_2_O in a ratio of 1:4, which was then mixed with a vortexer (Heidolph Instruments GmbH & C, Schwabach, Germany). 

#### 5.2.3. Chromatographic Methods

Aflatoxins were determined using HPLC with post-column derivatization and fluorescence detection (Ex: 360 nm, Em: 440 nm) preceded by post-column deprivatization (HPLC: L-7100 pump; L-7250 autosampler; L-7300 oven; L-7480, FLD L-7480 made by Merck (Hitachi, Darmstadt, Germany)). We used a chromatographic column: LiChroCART 250-4, LiChrospher 100 RP-18 (250 × 4 mm, 5 µm). The mobile phase was ACN:MeOH:H_2_O (20:20:60) + 119 mg KBr + 100 µL 65% HNO_3_ and the flow rate was 1 mL/min. Injection volume: 50 µL. 

Ochratoxin A was determined by using HPLC with fluorescence detection (Ex: 330 nm, Em: 460 nm), HPLC: LaChrom ELITE (Merk-Hitachi, Darmstadt, Germany). We used a chromatographic column (LiChropsher 100 RP-18) (250 × 4 mm, 5 µm), mobile phase: ACN: 2% CH_3_COOH (70:30). The flow rate was 1 mL/min and the injection volume was 50 µL.

Extractions of trichothecenes and zeralaenone were performed by HPLC with MS/MS detection. HPLC: Nexerra (Shimadzu (AB Sciex, Foster City, CA, USA)). The mass spectrometer was API 4000 (AB Sciex) and used columns Gemini C18NX (150 × 4.6 mm, 3 µm) (Phenomenex Inc., Torrance, CA, USA). The mobile phase was A: H_2_O + 5 mM CH_3_COONH_4_ + 1% CH_3_COOH, B: MeOH + 5 mM CH_3_COONH_4_ + 1% CH_3_COOH. The flow rate was 0.5 mL/min and the injection volume was up to 7 µL. Validation parameters are presented in Table 2. 

## Figures and Tables

**Table 1:** Total number of fungi, molds and yeasts in the analysed material.

	Total Number of Fungi (cfu/g)	Total Number of Molds (cfu/g)	Total Number of Yeast (cfu/g)
Positive samples/Number of samples	17/21	16/21	7/21
Max	6393	6303	1303
Mean	1108	1012	96

Max is the maximum number of colonies in one sample. Mean is the average of total colonies in the samples.

**Table 2:** Validation parameters for analyzed Mycotoxins [ng/g].

	DON	NIV	3ADON	MAS	DAS	T-2	HT-2	ZEN	OTA	AFB1	AFB2	AFG1	AFG2
LOD	1.6	2	2	1	0.7	0.3	1	0.16	0.13	0.05	0.02	0.15	0.08
LOQ	5	6	6	3	2	1	3	0.5	0.4	0.15	0.06	0.50	0.24
RSD	89 ± 9	79 ± 7	90 ± 6	87 ± 7	90 ± 5	90 ± 3	86 ± 7	98 ± 6	94 ± 4	85 ± 6	87 ± 11	83 ± 7	78 ± 5
R	500	250	250	125	125	250	250	125	25	10	2.5	10	2.5

LOD—detection limit; LOQ—quantification limit; RSD—standard deviation relative; R—recovery.

## Data Availability

Not applicable.
